# Inadequate Gestational Weight Gain and Exposure to Second-Hand Smoke during Pregnancy Increase the Risk of Low Birth Weight: A Cross-Sectional Study among Full-Term Infants

**DOI:** 10.3390/ijerph18031068

**Published:** 2021-01-26

**Authors:** Muliana Edi, Yit Siew Chin, Fui Chee Woon, Geeta Appannah, Poh Ying Lim, on behalf of the MICOS Research Group

**Affiliations:** 1 Department of Nutrition, Faculty of Medicine and Health Sciences, Universiti Putra Malaysia, Selangor 43400, Malaysia; muliana_edi@yahoo.com (M.E.); fuichee88@gmail.com (F.C.W.); geeta@upm.edu.my (G.A.); 2 Research Centre of Excellence, Nutrition and Non-Communicable Diseases, Faculty of Medicine and Health Sciences, Universiti Putra Malaysia, Selangor 43400, Malaysia; 3 Faculty of Engineering and Information Sciences, School of Mathematics and Applied Statistics, University of Wollongong, Wollongong, NSW 2522, Australia; 4 Department of Community Health, Faculty of Medicine & Health Sciences, Universiti Putra Malaysia, Selangor 43400, Malaysia; pohying_my@upm.edu.my

**Keywords:** low birth weight, infant, MICOS, gestational weight gain, exposure to second-hand smoke

## Abstract

Despite the advancement of the healthcare system, low birth weight (LBW) remains as one of the leading causes of under-five mortality. This cross-sectional study aimed to determine the prevalence of LBW and its associated factors among 483 third trimester pregnant women recruited from six selected public health clinics in the Federal Territory of Kuala Lumpur and the state of Selangor, Malaysia. Pregnant women were interviewed for information on socio-demographic characteristics, smoking behaviour, and second-hand smoke (SHS) exposure at home and in the workplace. Information on the obstetrical history and prenatal care visits history were retrieved from the maternal medical records, while infant’s birth outcomes were retrieved from infant medical records. The prevalence of LBW (<2.5 kg) in infants was 10.4%, with a mean birth weight of 3.0 [standard deviation (SD) 0.4] kg. Results from the multivariable logistic regression model showed that inadequate weight gained during pregnancy [odds ratio (OR) = 2.41, 95% confidence interval (CI) = 1.18–4.90] and exposure to SHS at home (OR = 1.92, 95% CI = 1.03–3.55) were significantly associated with LBW. In conclusion, pregnant women should monitor their rate of weight gain throughout pregnancy and avoid SHS exposure at home to reduce the risk of delivering LBW infants.

## 1. Introduction

Birth weight is a vital parameter that reflects maternal nutritional status and well-being before and during pregnancy, as well as foetal growth and development. LBW is defined as the weight taken at birth of less than 2.5 kg by the World Health Organization (WHO) [1]. LBW is a significant global public health concern and has been identified as one of the leading causes of mortality and morbidity among children under five years of age [2,3]. Globally, it is estimated that more than 20 million infants are born with LBW each year [4]. The prevalence of LBW varies across regions, ranging from 7.9% in the regions of Northern America to 17.3% in the regions of Asia [3]. LBW is more common in developing than developed countries, consisting up to 95.6% of the global prevalence [5]. In Malaysia, the third National Health and Morbidity Survey (NHMS III) revealed that 9.7% of the children under five years of age were born with LBW [6]. The prevalence of LBW in Malaysia varies across states, ranging from 5.7% to 16.2% [6].

LBW is associated with a range of short- and long-term consequences, and these consequences may persist into adulthood [7]. The “Barker’s hypothesis” or theory of “foetal and infant origins of adult disease” suggests that the in-utero environment and early infant health status can permanently program the growth and metabolism of the body, thereby influencing the development of chronic diseases in later life [8]. Evidence shows that LBW infants have a higher risk of getting illnesses, having congenital abnormalities, lower cognitive abilities, malnutrition, and impaired immune function, which increases the risk of infections [7,9,10,11,12]. Meanwhile, LBW was found to be associated with an increased risk of chronic diseases later in life including asthma, depression, type II diabetes, coronary heart disease, hypertension, stroke, insulin resistance, and cancers [7,13,14,15,16,17]. Considering both the short- and long-term consequences of LBW, it is therefore crucial to determine their risk factors, which may be targeted in future prevention strategies in order to reduce the risk of LBW in infants.

Findings from previous studies demonstrated that maternal factors including ethnicity, maternal age, educational level, employment status, monthly household income, household size, gravidity, and parity are important factors that are associated with LBW in infants [18,19,20,21,22,23,24,25]. Other factors including maternal pre-pregnancy body mass index (BMI), gestational weight gain (GWG), frequency of antenatal care visit, and environmental factors such as smoking behaviour and second-hand smoke (SHS) exposure during pregnancy are key modifiable risk factors of LBW [20,21,26,27,28]. Despite extensive research on LBW, findings remain inconclusive and there are limited studies assessing factors associated with LBW among full-term infants [29,30]. In addition, evidence on associations of the modifiable risk factors such as maternal pre-pregnancy BMI, GWG, and SHS exposure with LBW were mostly drawn from the developed countries [28,31,32,33]. In developing countries with a higher prevalence of overweight/obesity and smoking such as Malaysia, the contributions of maternal pre-pregnancy BMI, GWG, and SHS exposure on infant’s birth outcomes may be more pronounced. Although the adverse effects of tobacco smoke exposure on LWB have been well-established [28,30,33], studies that assessed the associations between SHS exposure at home and in the workplace during pregnancy with LBW have been limited [34,35].

Therefore, the present study aims to determine the prevalence of LBW and its associated maternal and environmental risk factors among full-term infants. Identifying the risk factors of LBW could help to guide the development of prevention strategies in order to reduce the incidence of LBW and indirectly reduce childhood mortality and morbidity resulting from LBW.

## 2. Materials and Methods

### 2.1. Study Setting and Population

This cross-sectional study is part of the Mother and Infant Cohort Study (MICOS) that focused on the contribution of early nutrition on the development of malnutrition and allergic diseases in infants during the first year of life, whereby this paper is focused on the risk factors associated with LBW among full-term infants. The protocol of MICOS has been described elsewhere [36]. The present study was conducted at six selected public health clinics in the Federal Territory of Kuala Lumpur and the state of Selangor, Malaysia, from November 2016 to January 2018. A total of 535 pregnant women aged ≥ 18 years, ≥28 weeks of gestations, with a singleton pregnancy, and who have their regular prenatal care visits at the selected public health clinics, were recruited. Women with multiple pregnancies and a preterm birth at less than 37 weeks gestation were excluded from the study. The present study was approved by the Medical Research and Ethics Committee (MREC), Ministry of Health (MOH) Malaysia (Reference number: NMRR-16-1047-30685), and the Ethics Committee for Research Involving Human Subjects, Universiti Putra Malaysia (JKEUPM) (Reference number: FPSK (FR16) P006). Prior to the data collection, written informed consent was obtained from the pregnant women. Out of the 535 pregnant women who consented, 52 of them were excluded due to preterm delivery (*n* = 11), incomplete maternal data (*n* = 3), and lack of data on birth outcomes (*n* = 38). The final sample size was 483 mother–infant pairs, with a response rate of 90.2%.

### 2.2. Maternal Characteristics

Information on socio-demographic characteristics including maternal age, ethnicity, educational level, employment status, household size, and monthly household income were obtained from the pregnant women through a face-to-face interview. Meanwhile, information on obstetrical history including pre-pregnancy weight, height, last measured weight at the third trimester of pregnancy, gravidity, and parity were obtained from the maternal medical records. Pre-pregnancy BMI (kg/m^2^) was calculated as pre-pregnancy weight (kg) divided by the square of pre-pregnancy height (m^2^) and was then classified into underweight (<18.5 kg/m^2^), normal weight (18.5–24.9 kg/m^2^), overweight (25.0–29.9 kg/m^2^), and obesity (≥30.0 kg/m^2^) [37]. Total GWG was calculated as the difference between the final recorded body weight at the last prenatal care visit and the pre-pregnancy weight recorded at the first prenatal care visit. Total GWG was then compared with the recommended range of weight gain based on their pre-pregnancy BMI using the 2009 Institute of Medicine (IOM) guidelines [38] and categorised into inadequate (gained weight less than the recommended range), adequate (gained weight within the recommended range), and excessive (gained weight more than the recommended range) GWG.

### 2.3. Prenatal Care Visit History

Information on prenatal care visit history including first prenatal care visit and the number of prenatal care visits were retrieved from the maternal medical records. First prenatal care visit was recorded as trimester of pregnancy in which the prenatal care was started, while the number of prenatal care visits was calculated as the total number of prenatal visits attended by the pregnant women (from the first attendance until the last attendance) at any primary healthcare facilities throughout their pregnancy.

### 2.4. Maternal Smoking and SHS Exposure during Pregnancy

Information on maternal smoking and SHS exposure during pregnancy were assessed using the Global Adult Tobacco Survey (GATS) Core Questionnaires developed by the Centre for Disease Control and Prevention (CDC) [39]. Maternal smoking during pregnancy (yes/no) was determined by the question “Do you currently smoke tobacco?” Exposure to SHS at home (yes/no) was defined as someone smoked inside the home of the pregnant women at least once a month, while exposure to SHS at the workplace (yes/no) was defined as someone smoked in indoor areas where the pregnant women work at least once a month.

### 2.5. Birth Outcomes

Information on birth outcomes, namely, gestational age at birth, infant’s sex, and birth weight, were extracted from the infant medical records. Low birth weight was defined as weight at birth of less than 2.5 kg [1]. Prior to the bivariate and multivariate analyses [40], the birth weight categories, such as very low birth weight and LBW, are merged as LBW and normal birth weight, whereas high birth weight is excluded.

### 2.6. Statistical Analysis

Data were analysed using IBM SPSS Statistics version 22.0 software (SPSS Inc. Chicago, IL, USA). All continuous variables were tested for normality and the value of skewness within the range of ±2.0 is considered as normally distributed [41]. Descriptive data for categorical variables were presented as frequencies and percentage, whereas continuous variables were presented as mean and standard deviation (SD). Simple logistic regression (SLR) analysis was used to determine the associations between each independent variable (maternal age, ethnicity, educational level, employment status, household size, monthly household income, pre-pregnancy BMI, total GWG, gravidity, parity, first prenatal care visit, number of prenatal care visits, maternal smoking during pregnancy, and exposure to SHS at home and at the workplace during pregnancy) with LBW, respectively. Variables with a *p*-value of less than 0.25 in the SLR model were included in the multivariable logistic regression model [42]. The forward variable selection method was used in the multivariable logistic regression analysis to determine the associations between the selected variables and LBW. All assumptions for the multivariate logistic regression analysis were met, in which all data were normally distributed, the *p*-value for the Omnibus test was less than 0.05, the percentage obtained in the overall classification table was more than 80.0%, the *p*-value for the Hosmer and Lemeshow test was greater than 0.05, and the *p*-value for multicollinearity and interaction was greater than 0.8 [40]. Results were presented as odds ratio (OR) and 95% confidence interval (CI) and the level of significance was set at *p* < 0.05.

## 3. Results

### 3.1. Characteristics of the Pregnant Women

Characteristics of the pregnant women are shown in Table 1. Overall, the mean age of the pregnant women was 30.0 (SD 4.2) years old (range: 18.6–40.7 years). Majority of them were Malay (91.1%), attained tertiary education (82.2%), were working (68.3%), had a moderate (52.6%) to high (30.2%) monthly household income, and had a mean household size of 3.9 (SD 1.9) members. While 9.7% of women were underweight before pregnancy, 36.9% of them were either overweight or obese before pregnancy. Additionally, more pregnant women had inadequate weight gained (32.7%) compared to those with excessive weight gained (28.2%) during pregnancy. The present study consisted of 0.8% of pregnant women who were smokers. Meanwhile, 32.4% of the pregnant women were exposed to SHS at home and 26.4% of them were exposed to SHS at their workplace. Majority of the pregnant women started their first prenatal care visit at the first trimester (71.6%), followed by the second (26.5%) and third (1.9%) trimester of pregnancy, with a mean gestation of 11.2 (SD 5.0) weeks. On average, the pregnant women attended approximately 11 prenatal care visits during their pregnancy.

### 3.2. Birth Outcomes

Birth outcomes of the full-term infants are presented in Table 2. The mean gestational age at birth was 38.4 (SD 1.3) weeks. Among 483 infants delivered, 50.5% of them were girls, and the remaining 49.5% were boys. Overall, 10.4% of the infants had LBW, with a mean birth weight of 3.0 (SD 0.4) kg.

### 3.3. Factors Associated with LBW

Results from the SLR analysis showed that low monthly household income (OR = 1.87, 95% CI = 0.23–0.97), inadequate gestational weight gain (OR = 2.28, 95% CI = 1.16–4.47), and being exposed to SHS at home (OR = 2.01, 95% CI = 1.09–3.69) were significantly associated with an increased risk of LBW (Table 3).

Variables significantly associated with LBW in the SLR analysis with a *p*-value less than 0.25 were included in a forward multivariate logistic regression model. These variables were educational level, household size, monthly household income, total GWG, gravidity, exposure to SHS at home, and total number of prenatal care visits. Results of the multivariate logistic regression model (Table 4) showed that pregnant women with inadequate GWG (OR = 2.41, 95% CI = 1.18–4.90) and exposed to SHS at home (OR = 1.92, 95% CI = 1.03–3.55) during pregnancy were associated with a higher risk of LBW. The multivariate logistic regression model was statistically significant (χ^2^ = 291.85, *p* = 0.004) and explained 5.8% of the variances in LBW.

## 4. Discussion

The present study revealed that about one in ten of the full-term infants were born with LBW. Inadequate GWG and exposure to SHS at home during pregnancy were the two main risk factors for LBW.

The prevalence of LBW reported in the present study was slightly higher than those reported in the NHMS III in Kuala Lumpur (7.2%) and Selangor (8.3%), Malaysia [6]. In contrast, our study reported a lower prevalence of LWB when compared to the global prevalence of LBW in the least developed countries (15.4%) [3] and other developing countries, including Indonesia (10.7%) [43], Nepal (12.0%) [21], and India (21.3%) [24]. Our study indicates that LBW remains an unsolved public health problem in Malaysia and continued efforts are needed to determine their modifiable risk factors which may be targeted in future prevention strategies.

The present study showed that pregnant women with inadequate weight gained during pregnancy were more likely to deliver an LBW infant. The association between inadequate GWG and a higher risk of LBW was also been observed among pregnant women with a normal pre-pregnancy BMI recruited from a government hospital in Turkey [32]. Similar findings were found in a retrospective study conducted among 7122 women in Slovakia that reported an increased risk of LBW among women with inadequate GWG [44]. The plausible underlying mechanism for the association between inadequate GWG and LBW remains unclear. Nutritional requirements increase during pregnancy to support both maternal and foetal growth. Inadequate GWG may reflect inadequate nutritional stores of the women during pregnancy, which failed to adequately satisfy the needs of both mother and foetus simultaneously. This could lead to maternal–foetal competition for available nutrients, and subsequently affects the birth weight of the infants [45,46].

Excessive GWG was prevalent in our study population. A significant positive association between GWG during pregnancy and birth weight was reported in previous studies [47,48,49], whereby a one kilogram increase in GWG was associated with about a 0.1 kg increase in birth weight [49]. Despite that, the present study did not observe a significant association between excessive GWG and LBW. Although the association was not significant, our results showed that 30.0% of the pregnant women who gained excessive weight during pregnancy delivered an LBW infant. It is possible that birth weight of the infants is not mainly influenced by GWG itself but is also influenced by other underlying factors which were not included in the present study, namely, food intake, physical activity, and the condition of placenta and amniotic fluid [38,50]. Therefore, there is a need to conduct further studies to determine other possible maternal factors and their confounders that are associated with LBW, especially the rate and pattern of GWG, as well as the condition of placenta and amniotic fluid. The examination of the associations between these factors and LBW is vital for the planning and enforcement of intervention programs and activities during prenatal care visits.

The present study shows that exposure to SHS at home was significantly associated with LBW. Consistent with the findings from several local studies in Malaysia, pregnant women who were exposed to SHS during pregnancy had a higher risk of delivering an LBW infant [35,51]. Bailey and colleagues reported that birth weight of the infants increased by about 0.4 kg when pregnant mothers refrained from being exposed to SHS [52]. The significant association between SHS at home and LBW can be explained by the adverse effects of SHS, in which the SHS contains multiple chemical components that are considered toxic to the foetus. These chemical components would impair the placenta and reduce the blood, oxygen, and nutrients being transferred to the foetus, which lead to growth restriction and hypoxia [49,53,54]. Inconsistent with previous studies, we found no significant associations between maternal smoking and exposure to SHS at the workplace during pregnancy with LBW [55,56]. Smoking was uncommon in our study populations and we found that only 0.8% of the women smoked during pregnancy. The small sample size for women who smoked during pregnancy in the present study may lead to a limited power to detect a statistically significant association. Although one in four of the pregnant women in the present study were exposed to SHS at their workplace, the smokers will usually smoke outside of the workplace. In the interim, most workplaces have enforced a law that prohibits smoking at the workplace, while some workplaces provide a smoking area for their employees who smoke.

Inconsistent with previous studies, we found no associations between any of the socio-demographic characteristics and obstetrical factors with LBW. It contradicted the findings from the previous studies in which pregnant women with older age, more household members, unemployed, and financially poor were associated with a higher risk of delivering an LBW infant [19,51]. Several local studies conducted among Malaysian pregnant women demonstrated that lower pre-pregnancy BMI, primigravida, and primiparous were associated with a higher risk of LBW [20,25,57]. The inconsistent results might be attributed to methodology differences across studies in terms of definitions for the variables, as well as the homogenous characteristics of pregnant mothers in the present study.

We found no significant association between prenatal care visit history and LBW in the present study, which contradicted the findings from previous studies that reported a lower risk of LBW among pregnant women who started their prenatal care visit earlier and had at least 4 prenatal care visits [21,51]. This contradicting finding might be attributed to the fact that pregnant women in the present study can fully utilise the knowledge or advice given during a series of face-to-face or group counselling services regarding lifestyle, pregnancy, and delivery during prenatal care visits provided by the health professionals at the primary healthcare institutions, even though the pregnant women had fewer prenatal care visits. Previous studies showed that the counselling services provided by healthcare professionals play an important role in reducing the occurrence of health problems and improving the growth and development of the foetus [58,59,60,61]. Besides, pregnant women in the present study might have access to various pregnancy monitoring applications and social media (e.g., WhatsApp’s, Instagram, and Facebook) that provide health information on pregnancy, which enable them to learn some nutrition knowledge and have invisible prenatal care visits to monitor the growth and development of their infants throughout pregnancy [62].

Several limitations need to be considered in the present study. Firstly, this was a cross-sectional study and the cause–effect relationships between the independent variables and LBW were unable to be determined. Secondly, this study was conducted among mother–infant pairs in Selangor and Kuala Lumpur, Malaysia, with an unbalanced proportion of ethnicity among respondents (91.1% Malay vs. 8.9% Non-Malay). Thus, findings from the present study may not be generalised to other populations. Thirdly, self-reported exposure to SHS may lead to underestimation or overestimation due to recall bias. Lastly, there is a discrepancy in the number of infants with LBW compared to those with normal birth weight. This discrepancy might reduce the power to detect some risk factors, for instance: socio-demographic characteristics (educational level, employment status, household size, and monthly household income), obstetrical history (pre-pregnancy BMI, gravidity, and parity), and prenatal care visit history (first prenatal care visit and number of prenatal care visits).

## 5. Conclusions

To conclude, one in ten of the full-term infants were born with LBW. Inadequate GWG and exposure to SHS at home during pregnancy were associated with a higher risk of LBW in infants. The present study suggests that women should monitor their rate of weight gain throughout pregnancy and avoid SHS exposure at home in order to reduce the risk of delivering an LBW infant. In addition, pregnant women should be informed about the importance of gaining sufficient weight throughout pregnancy and the adverse effects of SHS exposure at home during their routine prenatal care visit.

## Figures and Tables

**Table 1:** Characteristics of the pregnant mothers (*n* = 483).

Variables	*n* (%)	Mean (SD)
**Socio-demographic Characteristics**		
**Maternal age (years)**		30.0 (4.2)
**Ethnicity**		
Malay	440 (91.1)	
Chinese	26 (5.4)	
Indian	11 (2.3)	
Others	6 (1.2)	
**Educational level**		
Secondary	86 (17.8)	
Tertiary	397 (82.2)	
**Employment status**		
Not working	153 (31.7)	
Working	330 (68.3)	
**Household size (member)**		3.9 (1.9))
**Monthly household income (RM)** ^a^		
Low (<RM2300)	83 (17.2)	
Moderate (RM2300–RM5599)	254 (52.6)	
High (>RM5599)	146 (30.2)	
**Obstetrical History**		
**Pre-pregnancy weight (kg)**		59.3 (13.4)
**Pre-pregnancy height (cm)**		156.7 (5.7)
**Pre-pregnancy BMI (kg/m^2^)**		24.1 (4.9)
Underweight (<18.5)	47 (9.7)	
Normal (18.5–24.9)	258 (53.4)	
Overweight (25.0–29.9)	124 (25.7)	
Obesity (≥30.0)	54 (11.2)	
**Total gestational weight gain (kg)**		12.2 (5.1)
Inadequate	158 (32.7)	
Adequate	189 (39.1)	
Excessive	136 (28.2)	
**Gravidity**		1.5 (1.5)
Primigravida	169 (35.0)	
Multigravida	314 (65.0)	
**Parity**		1.0 (1.2)
Nulliparous	205 (42.4)	
Primiparous	125 (25.9)	
Multiparous	153 (31.7)	
**Maternal smoking during pregnancy**		
Yes	4 (0.8)	
No	479 (99.2)	
**Exposure to SHS at home (*n* = 472) ^b^**		
Yes	153 (32.4)	
No	319 (67.6)	
**Exposure to SHS at workplace (*n* = 330) ^c^**		
Yes	87 (26.4)	
No	243 (73.6)	
**Prenatal Care Visit History**		
**First prenatal care visit (week)**		11.2 (5.0)
First trimester	346 (71.6)	
Second trimester	128 (26.5)	
Third trimester	9 (1.9)	
**Total number of prenatal care visits**		10.8 (3.0)

Note: RM: Ringgit Malaysia, BMI: Body mass index, SD: standard deviation. ^a^ 1 US dollar = RM 4.19 (as of 15 August 2020). ^b^ Sample size for exposure of second-hand smoke (SHS) at home varied (*n* = 472) because six pregnant mothers were divorced and unmarried. ^c^ Sample size for exposure of SHS at the workplace varied (*n* = 330) because 153 pregnant mothers were not working.

**Table 2:** Birth outcomes of the infants (*n* = 483).

Birth Outcomes	*n* (%)	Mean (SD)
**Gestational age at birth (week)**		38.4 (1.3)
**Sex**		
Boy	239 (49.5)	
Girl	244 (50.5)	
**Birth weight (kg)**		3.0 (0.4)
Extremely Low (<1.0)	0 (0.0)	
Very Low (<1.5)	3 (0.6)	
Low (<2.5)	47 (9.7)	
Normal (2.5–4.0)	428 (88.6)	
High (>4.0)	5 (1.1)	

**Table 3:** Simple logistic analysis of maternal factors and low birth weight (LBW) (*n* = 478).

Variables	LBW (*n* = 50)	Normal (*n* = 428)	OR	95% CI	*p*-Value
**Socio-demographic Characteristics**					
**Age (years)**					
Mean (SD)	30.3 (4.8)	29.9 (4.1)	1.020	0.95–1.09	0.566
**Ethnicity**					
Malay	46 (92.0)	390 (91.1)	Reference	-	-
Chinese	1 (2.0)	25 (5.8)	0.339	0.05–2.56	0.295
Indian	2 (4.0)	8 (1.9)	2.120	0.44–10.28	0.351
Others	1 (2.0)	5 (1.2)	1.696	0.19–14.83	0.633
**Educational level**					
Secondary	14 (28.0)	72 (16.8)	Reference	-	-
Tertiary	36 (72.0)	356 (83.2)	0.520	0.27–1.01	0.055
**Employment status**					
Not working	18 (36.0)	134 (31.3)	Reference	-	-
Working	32 (64.0)	294 (68.7)	0.810	0.44–1.50	0.501
**Household size** (member)					
Mean (SD)	3.6 (1.3)	4.0 (2.0)	0.898	0.76–1.07	0.226
**Monthly household income** ^a^					
Low (<RM2300)	14 (28.0)	69 (16.1)	1.870	0.23–0.97	0.041 *
Moderate (RM2300–RM5599)	22 (44.0)	230 (53.7)	0.881	0.84–4.15	0.124
High (>RM5599)	14 (28.0)	129 (30.1)	Reference	-	-
**Obstetrical History**					
**Pre-pregnancy BMI**					
Underweight (<18.5 kg/m^2^)	6 (12.0)	40 (9.3)	1.283	0.50–3.31	0.605
Normal (18.5–24.9 kg/m^2^)	27 (54.0)	231 (54.0)	Reference	-	-
Overweight (25.0–29.9 kg/m^2^)	11 (22.0)	112 (26.2)	0.840	0.40–1.76	0.643
Obesity (≥30.0 kg/m^2^)	6 (12.0)	45 (10.5)	1.141	0.45–2.92	0.784
**Total gestational weight gain** (kg)					
Inadequate	26 (52.0)	131 (30.6)	2.276	1.16–4.47	0.017 *
Adequate	15 (30.0)	172 (40.2)	Reference	-	-
Excessive	9 (18.0)	125 (29.2)	0.826	0.35–1.95	0.662
**Gravidity**					
Primigravida	12 (24.0)	157 (36.7)	Reference	-	-
Multigravida	38 (76.0)	271 (63.3)	1.835	0.93–3.61	0.079
**Parity**					
Nulliparous	19 (38.0)	185 (43.2)	Reference	-	-
Primiparous	14 (28.0)	109 (25.5)	1.251	0.60–2.60	0.548
Multiparous	17 (34.0)	134 (31.3)	1.235	0.62–2.47	0.549
**Maternal smoking during pregnancy**					
Yes	0 (0.0)	4 (0.9)	<0.001	<0.001–<0.001	0.999
No	50 (100.0)	424 (99.1)	Reference	-	-
**Exposure to SHS at home** (*n* = 472) ^b^					
Yes	22 (46.8)	128 (30.5)	2.007	1.09–3.69	0.025 *
No	25 (53.2)	292 (69.5)	Reference		
**Exposure to SHS at workplace** (*n* = 330) ^c^					
Yes	10 (34.5)	77 (18.0)	1.517	0.68–3.41	0.312
No	19 (65.5)	222 (51.9)	Reference	-	-
**Prenatal care visit history**					
**First prenatal care visit**					
First trimester	39 (78.0)	303 (70.8)	Reference	-	-
Second trimester	10 (20.0)	117 (27.3)	0.664	0.32–1.37	0.270
Third trimester	1 (2.0)	8 (1.9)	0.971	0.12–7.97	0.978
**Total number of prenatal care visits**					
Mean (SD)	10.2 (2.5)	10.9 (3.0)	0.931	0.84–1.03	0.159

* *p* < 0.05. ^a^ 1 US dollar = RM 4.19 (as of 15 August 2020). ^b^ Sample size for exposure of SHS at home varied (*n* = 472) because six pregnant mothers were divorced and unmarried. ^c^ Sample size for exposure of SHS at workplace varied (*n* = 330) because 153 pregnant mothers were not working. OR: odds ratio, CI: confidence interval.

**Table 4:** Factors associated with LBW in full-term infants (*n* = 478).

Characteristics of the Pregnant Mothers	OR	95% CI	*p*-Value
**Total gestational weight gain (kg)**			
Adequate	Reference		
Inadequate	2.41	1.18–4.90	0.016 *
Excessive	0.95	0.39–2.31	0.916
**Exposure to SHS at home**			
Yes	1.92	1.03–3.55	0.039 *
No	Reference		

Multivariate logistic regression model: χ^2^ = 291.85, *p* = 0.004, Nagelkerke R square = 0.058, Cox and Snell R square = 0.028, Prediction accuracy based on overall classification table = 89.9%, Hosmer and Lemeshow test = 0.454. * *p* < 0.05.

## References

[1] World Health Organisation (WHO) (2010). International Statistical Classification of Diseases and Related Health Problems.

[2] World Health Organisation (WHO) Sexual and Reproductive Health: More Women Worldwide Receive Early Antenatal Care, but Great Inequalities Remain. https://www.who.int/reproductivehealth/early-anc-worldwide/en/.

[3] United Nations Children’s Fund (UNICEF), World Health Organization (WHO) (2019). UNICEF-WHO Low Birthweight Estimates: Levels and Trends 2000–2015.

[4] World Health Organisation (WHO) (2014). WHA Global Nutrition Targets 2025: Low Birth Weight Policy Brief.

[5] World Health Organization (WHO), United Nations Children’s Fund (UNICEF) (2004). Low Birthweight: Country, Regional and Global Estimates.

[6] Institute for Public Health (IPH), National Institutes of Health (NIH), Ministry of Health Malaysia (2016). National Health and Morbidity Survey (NHMS) 2016—Vol II: Maternal and Child. Health Findings.

[7] Negrato C.A., Gomes M.B. (2013). Low birth weight: Causes and consequences. Diabetol. Metab. Syndr..

[8] Barker D.J.P. (2001). Fetal and infant origins of adult disease. Mon. Kinderheilkd.

[9] Valla L., Wentzel-Larsen T., Hofoss D., Slinning K. (2015). Prevalence of suspected developmental delays in early infancy: Results from a regional population-based longitudinal study. BMC Pediatr..

[10] Kingston D., Heaman M., Brownell M., Ekuma O. (2015). Predictors of childhood anxiety: A population-based cohort study. PLoS ONE.

[11] Pascoe L., Roberts G., Doyle L.W., Lee K.J., Thompson D.K., Seal M.L., Josev E.K., Nosarti C., Gathercole S., Anderson P.J. (2013). Preventing academic difficulties in preterm children: A randomised controlled trial of an adaptive working memory training intervention—IMPRINT study. BMC Pediatr..

[12] Drozd-Dabrowska M., Trusewicz R., Ganczak M. (2018). Selected risk factors of developmental delay in Polish infants: A case-control study. Int. J. Environ. Res. Public Health.

[13] Kinge J.M. (2017). Variation in the relationship between birth weight and subsequent obesity by household income. Health Econ. Rev..

[14] Triebwasser C., Wang R., DeWan A.T., Metayer C., Morimoto L., Wiemels J.L., Kadan-Lottick N., Ma X. (2016). Birth weight and risk of paediatric Hodgkin lymphoma: Findings from a population-based record linkage study in California. Eur. J. Cancer.

[15] Liu X., Olsen J., Agerbo E., Yuan W., Cnattingius S., Gissler M., Li J. (2015). Birth weight, gestational age, fetal growth and childhood asthma hospitalization. Int. J. Epidemiol..

[16] Spracklen C.N., Wallace R.B., Sealy-Jefferson S., Robinson J.G., Freudenheim J.L., Wellons M.F., Saftlas A.F., Snetselaar L.G., Manson J.E., Hou L. (2014). Birth weight and subsequent risk of cancer. Cancer Epidemiol..

[17] Risnes K.R., Vatten L.J., Baker J.L., Jameson K., Sovio U., Kajantie E., Osler M., Morley R., Jokela M., Painter R.C. (2011). Birthweight and mortality in adulthood: A systematic review and meta-analysis. Int. J. Epidemiol..

[18] Young R.L., Declercq E. (2009). Implications of subdividing marital status: Are unmarried mothers with partners different from unmarried mothers without partners? An exploratory analysis. Matern. Child. Health J..

[19] Yadav H., Lee N. (2013). Maternal factors in predicting low birth weight babies. Med. J. Malays..

[20] Karim M.R., Mondal M.N.I., Rana M.M., Karmaker H., Bharati P., Hossain M.G. (2016). Maternal factors are important predictors of low birth weight: Evidence from Bangladesh demographic & health survey-2011. Malays. J. Nutr..

[21] Bhaskar R.K., Deo K.K., Neupane U., Chaudhary Bhaskar S., Yadav B.K., Pokharel H.P., Pokharel P.K. (2015). A case control study on risk factors associated with low birth weight babies in Eastern Nepal. Int. J. Pediatr..

[22] Solanke B.L., Bisiriyu A.L., Akinlo A. (2018). Socio-demographic correlates of child size at birth in Nigeria. IRPG.

[23] Dasgupta A., Basu R. (2011). Determinants of low birth weight in a Block of Hooghly, West Bengal: A multivariate analysis. Int. J. Biol. Med. Res..

[24] Kumari A., Kumar S. (2020). A study on maternal factors affecting low birth weight in institutional deliveries at IGMS, Patna. Int. J. Sci. Res..

[25] Kaur S., Ng C.M., Badon S.E., Jalil R.A., Maykanathan D., Yim H.S., Jan Mohamed H.J. (2019). Risk factors for low birth weight among rural and urban Malaysian women. BMC Public Health.

[26] Nurfazlin R., Hayati Adilin M.A.M., Siti Shafura A., Ajau D., Khairil Anuar M.I. (2012). The association of gestational weight gain and the effect on pregnancy outcome defined by BMI group among women delivered in Hospital Kuala Lumpur, Malaysia: A retrospective study. Asian J. Clin. Nutr..

[27] Coelho N.d.L.P., Cunha D.B., Esteves A.P.P., Lacerda E.M.d.A., Theme Filha M.M. (2015). Dietary patterns in pregnancy and birth weight. Rev. Saude Publica.

[28] Khader Y.S., Al-Akour N., Alzubi I.M., Lataifeh I. (2011). The association between second hand smoke and low birth weight and preterm delivery. Matern. Child Health J..

[29] Gebregzabiherher Y., Haftu A., Weldemariam S., Gebrehiwet H. (2017). The prevalence and risk factors for low birth weight among term newborns in Adwa General Hospital, Northern Ethiopia. Obstet. Gynecol. Int..

[30] Wahabi H.A., Alzeidan R.A., Fayed A.A., Mandil A., Al-Shaikh G., Esmaeil S.A. (2013). Effects of secondhand smoke on the birth weight of term infants and the demographic profile of Saudi exposed women. BMC Public Health.

[31] Lima R., Batista R.F.L., Ribeiro M.R.C., Ribeiro C.C.C., Simoes V.M.F., Lima Neto P.M., Silva A., Bettiol H. (2018). Prepregnancy body mass index, gestational weight gain, and birth weight in the BRISA cohort. Rev. Saude Publica.

[32] Eraslan Sahin M., Col Madendag I. (2019). Effect of gestational weight gain on perinatal outcomes in low risk pregnancies with normal prepregnancy body mass index. Biomed. Res. Int..

[33] Windham G.C., Hopkins B., Fenster L., Swan S.H. (2000). Prenatal active or passive tobacco smoke exposure and the risk of preterm delivery or low birth weight. Epidemiology.

[34] Krishnamurthy A.V., Chinnakali P., Dorairajan G., Sundaram S.P., Sarveswaran G., Sivakumar M., Krishnamoorthy K., Dayalane H., Sinouvassan V. (2018). Tobacco use, exposure to second-hand smoke among pregnant women and their association with birth weight: A retrospective cohort study. J. Fam. Med. Prim. Care.

[35] Norsa‘adah B., Salinah O. (2014). The effect of second-hand smoke exposure during pregnancy on the newborn weight in Malaysia. Malays. J. Med. Sci..

[36] Woon F.C., Chin Y.S., Ismail I.H., Chan Y.M., Batterham M., Abdul Latiff A.H., Gan W.Y., Appannah G. (2018). Contribution of early nutrition on the development of malnutrition and allergic diseases in the first year of life: A study protocol for the Mother and Infant Cohort Study (MICOS). BMC Pediatr..

[37] World Health Organisation (WHO) (2000). Obesity: Preventing and Managing the Global Epidemic: Report of a WHO Consultation.

[38] Institute of Medicine (IOM) (2009). Weight Gain during Pregnancy: Reexamining the Guidelines.

[39] Global Adult Tobacco Survey Collaborative Group (2011). Tobacco Questions for Surveys: A Subset of Key Questions from the Global Adult Tobacco Survey (GATS).

[40] Reimann C., Filzmoser P., Garrett R.G., Dutter R. (2008). Statistical Data Analysis Explained: Applied Environmental Statistics with R.

[41] Hosmer D.W., Lemeshow S. (2000). Applied Logistic Regression.

[42] Allen P., Bennett K., Heritage B. (2014). SPSS Statistics Version 22: A Practical Guide.

[43] Kartasurya M.I., Dharmawan Y., Widjanarko B., Handayani N. (2017). The prediction model for low birth weight in Batang district, Central Java, Indonesia. Adv. Sci. Lett..

[44] Simko M., Totka A., Vondrova D., Samohyl M., Jurkovicova J., Trnka M., Cibulkova A., Stofko J., Argalasova L. (2019). Maternal body mass index and gestational weight gain and their association with pregnancy complications and perinatal conditions. Int. J. Environ. Res. Public Health.

[45] Scholl T.O., Hediger M.L., Schall J.I., Khoo C.S., Fischer R.L. (1994). Maternal growth during pregnancy and the competition for nutrients. Am. J. Clin. Nutr..

[46] Kramer M.S. (1987). Determinants of low birth weight: Methodological assessment and meta-analysis. Bull. World Health Organ..

[47] Restall A., Taylor R.S., Thompson J.M., Flower D., Dekker G.A., Kenny L.C., Poston L., McCowan L.M. (2014). Risk factors for excessive gestational weight gain in a healthy, nulliparous cohort. J. Obes..

[48] Chen Z., Du J., Shao L., Zheng L., Wu M., Ai M., Zhang Y. (2010). Prepregnancy body mass index, gestational weight gain, and pregnancy outcomes in China. Int. J. Gynaecol. Obstet..

[49] Tela F.G., Bezabih A.M., Adhanu A.K. (2019). Effect of pregnancy weight gain on infant birth weight among mothers attending antenatal care from private clinics in Mekelle City, Northern Ethiopia: A facility based follow-up study. PLoS ONE.

[50] Widen E.M., Factor-Litvak P.R., Gallagher D., Paxton A., Pierson R.N., Heymsfield S.B., Lederman S.A. (2015). The pattern of gestational weight gain is associated with changes in maternal body composition and neonatal size. Matern. Child Health J..

[51] Mohd Zain N., Low W.Y., Othman S. (2015). Impact of maternal marital status on birth outcomes among young Malaysian women: A prospective cohort study. Asia Pac. J. Public Health.

[52] Bailey B.A., McCook J.G., Hodge A., McGrady L. (2012). Infant birth outcomes among substance using women: Why quitting smoking during pregnancy is just as important as quitting illicit drug use. Matern. Child. Health J..

[53] Centers for Disease Control and Prevention, National Center for Chronic Disease Prevention and Health Promotion, Office on Smoking and Health (2010). How Tobacco Smoke Causes Disease: The Biology and Behavioral Basis for Smoking-Attributable Disease: A Report of the Surgeon General.

[54] de Machado J.B., Plinio Filho V.M., Petersen G.O., Chatkin J.M. (2011). Quantitative effects of tobacco smoking exposure on the maternal-fetal circulation. BMC Pregnancy Childbirth.

[55] Ko T.J., Tsai L.Y., Chu L.C., Yeh S.J., Leung C., Chen C.Y., Chou H.C., Tsao P.N., Chen P.C., Hsieh W.S. (2014). Parental smoking during pregnancy and its association with low birth weight, small for gestational age, and preterm birth offspring: A birth cohort study. Pediatr. Neonatol..

[56] Kataoka M.C., Carvalheira A.P.P., Ferrari A.P., Malta M.B., de Barros Leite Carvalhaes M.A., de Lima Parada C.M.G. (2018). Smoking during pregnancy and harm reduction in birth weight: A cross-sectional study. BMC Pregnancy Childbirth.

[57] Nazari M., Zainiyah S.Y., Lye M.S., Zalilah M.S., Heidarzadeh M. (2013). Comparison of maternal characteristics in low birth weight and normal birth weight infants. East. Mediterr. Health J..

[58] World Health Organisation (WHO) New Guidelines on Antenatal Care for a Positive Pregnancy Experience. https://www.who.int/reproductivehealth/news/antenatal-care/en/.

[59] Sullivan T.R., Hirst J.E. (2011). Reducing maternal mortality: A review of progress and evidence-based strategies to achieve millennium development goal 5. Health Care Women Int..

[60] World Health Organisation (WHO) (2003). Antenatal Care in Developing Countries: Promises, Achievements and Missed Opportunities. An. Analysis of Trends, Levels and Differentials.

[61] Carroli G., Rooney C., Villar J. (2001). How effective is antenatal care in preventing maternal mortality and serious morbidity? An overview of the evidence. Paediatr. Perinat. Epidemiol..

[62] Muula A.S., Siziya S., Rudatsikira E. (2011). Parity and maternal education are associated with low birth weight in Malawi. Afr. Health Sci..

